# U. S. V. M. A.

**Published:** 1897-05

**Authors:** 


### U. S. V. M. A.

STATE SECRETARIES, 1896–'97.—Arizona, D. LeMay, Fort Grant; Arkansas, R. R. Dinwiddie, Fayetteville; California, W. F. Egan, 1117 Golden Gate Avenue, San Francisco; Colorado, E. Pouppirt, Duff; Massachusetts, W. L. LaBaw, 50 Village Street, Boston; New Hampshire, L. Pope, Jr., 101 State Street, Portsmouth; Connecticut, R. P. Lyman, 328 Asylum Street, Hartford; Maine, William S. Lord, U. S. Hotel, Portland; Rhode Island, Walter L. Burt, 26 Tabor Avenue, Providence; New York, J. Faust, 209 Union Street, Poughkeepsie; New Jersey, D. J. Dixon, 366 Washington Street, Hoboken; Pennsylvania, S. J. J. Harger, 205 North Twentieth Street, Philadelphia; Delaware, H. P. Eves, 507 West Ninth Street, Wilmington; Maryland, William Dougherty, 1035 Cathedral Street, Baltimore; Virginia, J. P. Turner, Fort Meyer; North Carolina, F. P. Williamson, 122 West Hargett Street, Raleigh; South Carolina, Benjamin McInnis, Charleston; Alabama, C. A. Cary, Auburn; Mississippi, T. S. Butler, Agricultural College; Louisiana, W. H. Dalrymple, 207 North Boulevard, Baton Rouge; Texas, G. E. Griffin, Fort Sam Houston, San Antonio; Ohio, W. H. Gribble, Washington Court House; Indiana, T. J. Turner, Indianapolis, care Kingan & Co.; Michigan, S. Brenton, 83 Fifth Street, Detroit; Wisconsin, G. E. Leech, 150 Seventh Street, Milwaukee; Illinois, R. P. Steddom, Galesburg; Kentucky, John Graesll, 19 East Eleventh Street, Covington; West Virginia, L. N. Reefer, 1406 Chapline Street, Wheeling; District of Columbia, J. H. Adamson, Washington; Tennessee, W. C. Rayen, 529 Broad Street, Nashville; Iowa, G. A. Johnson, City Building, Sioux City; Minnesota, M. H. Reynolds, St. Anthony Park; Washington, S. B. Nelson, Pullman; North Dakota, T. D. Hinebauch, Fargo; South Dakota, M. J. Treacy, Fort Meade; Utah, Paul Fischer, Logan; Montana, M. E. Knowles, Butte; Kansas, N. S. Mayo, Manhattan; Missouri, J. M. Phillips, 3466 Eastern Avenue, St. Louis; Nebraska, A. T. Peters, Lincoln, Experiment Station.

---

### PRACTICE FOR SALE.

A large and well-paying veterinary practice, with feeding and boarding-stable attached to a large and well-equipped hospital, will be sold to the right kind of a veterinarian, who has the ambition and desire to procure a well-paying practice (will pay for itself in one year) in a beautiful little city in New York State, with no opposition of any importance. The present owner must sell out on account of ill health. For full particulars, address "Veterinarian," in care of DR. N. P. HINKLEY, 395 Ellicott Street, Buffalo, N. Y.

